# IL-8 signaling is involved in resistance of lung carcinoma cells to erlotinib

**DOI:** 10.18632/oncotarget.9662

**Published:** 2016-05-27

**Authors:** Romaine I. Fernando, Duane H. Hamilton, Charli Dominguez, Justin M. David, Kristen K. McCampbell, Claudia Palena

**Affiliations:** ^1^ Laboratory of Tumor Immunology and Biology, Center for Cancer Research, National Cancer Institute, NIH, Bethesda, MD, USA

**Keywords:** erlotinib, tumor resistance, IL-8, epithelial-mesenchymal transition

## Abstract

A signaling pathway that is frequently deregulated in human carcinomas and has been explored as a therapeutic target involves the activation of the epidermal growth factor receptor (EGFR). Inhibition of EGFR via the small molecule inhibitors erlotinib and gefitinib commonly results in tumor resistance, even in patients with EGFR-mutant tumors that initially show substantial clinical responses. This study was designed to broaden our understanding of the molecular mechanisms of acquired resistance to erlotinib in lung cancer cells bearing wild type or mutated EGFR. We report here that generation of erlotinib-resistant lung cancer cells *in vitro* resulted in a phenotypic alteration reminiscent of an epithelial-mesenchymal transition (EMT) concomitant with a robust upregulation of the IL-8/IL-8R axis. Our results also demonstrate that upregulation of p38 MAPK signaling is responsible for the enhanced IL-8 secretion in the erlotinib-resistant tumor cells. Blockade of IL-8 signaling effectively reduced mesenchymal features of the resistant cells and also markedly enhanced their susceptibility to erlotinib. These results provide a rationale for the development of new therapeutic approaches involving blockade of IL-8 signaling for the management of acquired resistance to EGFR inhibition in patients with lung cancer.

## INTRODUCTION

Acquisition of drug resistance is a common phenomenon observed during tumor progression. Refractoriness to anti-cancer treatment can be observed in response to a variety of therapies, including conventional chemotherapy, radiation, and some targeted therapies. A signaling pathway that is frequently deregulated in human carcinomas and has been intensively explored as a therapeutic cancer target involves the activation of the epidermal growth factor receptor (EGFR) via amplification or mutation [1]. Activation of EGFR is implicated in a variety of tumor types, including lung, colon, pancreas, and bladder cancer [2]. In the case of non-small cell lung carcinoma (NSCLC), the presence of defined mutations in the EGFR kinase domain [3, 4], known as sensitizing mutations, substantially increases the response to treatment with the EGFR-specific small molecule inhibitors erlotinib and gefitinib [5–7]. Although patients carrying these mutations respond favorably to EGFR inhibition, tumor relapse is common within one to two years of treatment due to acquired resistance to therapy [8]. In lung cancer patients with wild type EGFR tumors, EGFR inhibition could also be used in the second or third line of treatment [9] with modest and variable clinical responses. In those patients, acquisition of resistance to EGFR inhibition would also pose a significant challenge to treatment decisions.

Understanding the mechanisms involved in acquired resistance to EGFR inhibition has been a subject of intense investigation [10]. In approximately half of patients, tumor resistance is associated with the acquisition of a secondary EGFR mutation (Thr790Met modification) [8, 11]. Other mechanisms have been described in the remaining patients, including enhanced expression of c-MET [12, 13], activation of the nuclear factor-κB (NF-κB) pathway [14], or activation of the AXL kinase [15]. In a subset of patients, acquisition of resistance to EGFR inhibitors is associated with the phenomenon of epithelial-mesenchymal transition (EMT) [16–18], a plastic phenotypic conversion that allows carcinoma cells to acquire features typically associated with the mesenchymal phenotype [19, 20], including the ability to disseminate and to resist cell death [21, 22].

This study was designed to broaden our understanding of the molecular mechanisms leading to the acquired resistance to erlotinib in NSCLC cells bearing wild type or mutated EGFR. We report on the observation of a phenotypic alteration resembling the EMT occurring in erlotinib-resistant lung cancer cells. In agreement with our previous studies demonstrating a role for IL-8 signaling in the induction and maintenance of mesenchymal features in carcinoma cells [23, 24], the erlotinib-resistant cell lines generated in this study showed a robust upregulation of the IL-8/IL-8R axis as exhibited by enhanced secretion of IL-8 and enhanced levels of IL-8 receptor alpha (IL-8RA, CXCR1) expression. Upregulation of the IL-8 axis was concurrent to the activation of the mitogen-activated protein kinase p38, which is known to stabilize IL-8 mRNA. Blockade of IL-8 signaling with a neutralizing anti-IL-8 antibody was able to effectively drive the phenotypic reversion of the resistant cells while markedly enhancing their sensitivity to erlotinib. These results thus provide a rationale for the development of new therapeutic approaches involving blockade of IL-8 signaling for the management of acquired resistance to EGFR inhibition in patients with NSCLC.

## RESULTS

### Erlotinib resistance is associated with acquisition of mesenchymal tumor traits

**Figure 1:**
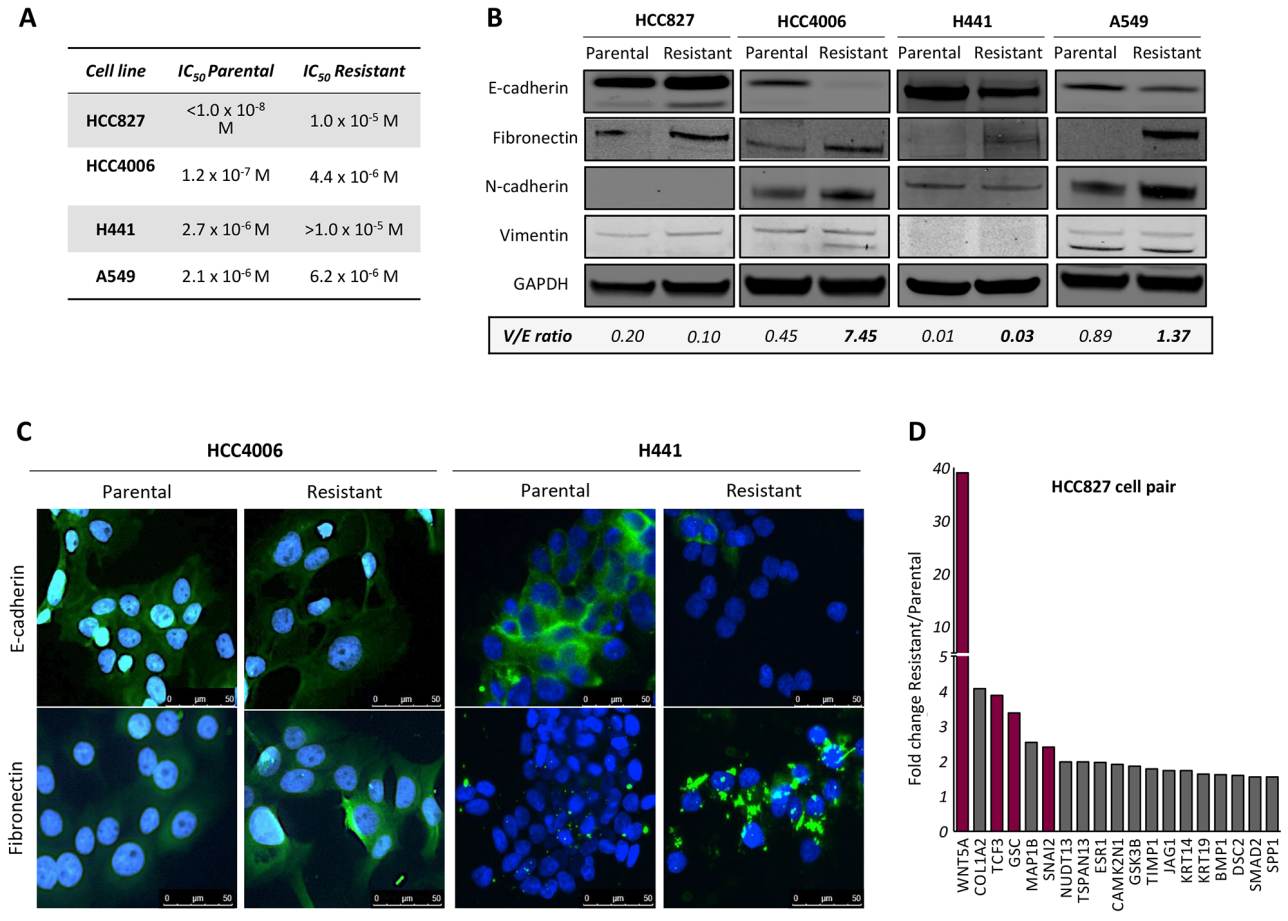
Resistance to erlotinib in NSCLC cells is associated with EMT **A.** Calculated IC_50_ values for parental and erlotinib-resistant tumor cell lines. **B.** Western blot analysis of protein lysates from indicated tumor cell pairs. Indicated below is the ratio of vimentin to E-cadherin (V/E) for each cell line (normalized to GAPDH). **C.** Representative images of HCC4006 and H441 parental vs. erlotinib-resistant cells assayed by immunofluorescence analysis of epithelial E-cadherin and mesenchymal fibronectin (green signal) in tumor cell pairs. Blue signal corresponds to DAPI stained nuclei. **D.** Fold change expression of EMT-related genes in resistant vs. parental HCC827 cells determined by real time PCR expression.

Non-small cell lung carcinoma (NSCLC) cells resistant to erlotinib were generated and maintained as described in the Materials and Methods section. Four cell lines were used in this study: HCC4006, HCC827 (EGFR exon 19 del), H441 and A549 (wild type EGFR, KRAS mutant). The IC_50_ values for the parental and corresponding resistant cell lines are shown in Figure 1A; all erlotinib-resistant lines showed a significant survival advantage compared to their parental counterparts, with IC_50_ values ranging from 4.4 to >10 μM. Phenotypic changes resembling an EMT were observed in the resistant cells, though the magnitude and pattern of changes varied among the lines. A significant reduction of epithelial E-cadherin and a marked increase of the mesenchymal markers fibronectin and/or N-cadherin were observed in the erlotinib-resistant lines derived from H441, A549, and HCC4006 cells (Figure 1B and 1C). In addition, the ratio of mesenchymal vimentin to epithelial E-cadherin (V/E ratio) was enhanced in 3 out of 4 erlotinib-resistant cells, compared to the parental counterparts. As shown in Figure 1B, no changes in E-cadherin, fibronectin, N-cadherin or vimentin were observed with HCC827 cells exposed to escalating concentrations of erlotinib in culture. As previous reports in the literature have demonstrated the ability of erlotinib or gefitinib to induce EMT in HCC827 cultures, we conducted analysis at the mRNA level. A marked upregulation of several mRNAs encoding for proteins associated with an EMT was observed with HCC827 erlotinib-resistant cells (Figure 1D). In particular, erlotinib-resistant HCC827 cells exhibited a substantial (~40-fold) upregulation of mRNA encoding for Wnt5a, a ligand of the non-canonical Wnt pathway that has been previously associated with EMT [25], as well as increased expression of mRNAs encoding for the EMT-transcriptional regulators Slug (*SNAI2*), goosecoid (*GSC*), and E2A (*TCF3*). These results indicated that HCC827 cells resistant to erlotinib also exhibited at least some of the changes characteristic of an EMT.

### Acquired erlotinib resistance is characterized by high levels of phosphorylated p38

**Figure 2:**
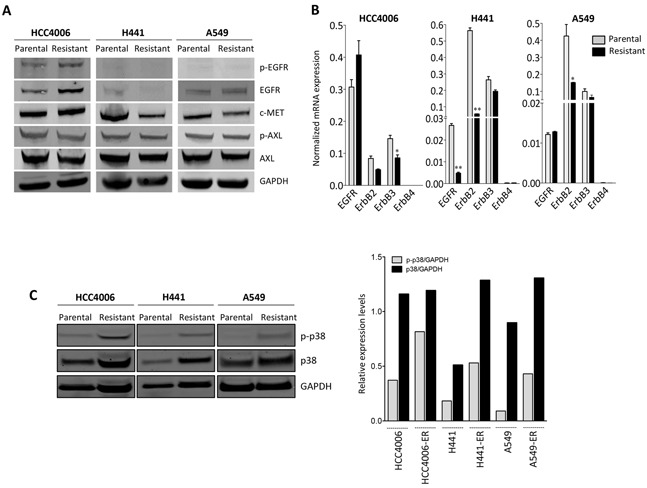
Upregulation of p38 signaling in erlotinib-resistant cells **A.** Western blot analysis of protein lysates from erlotinib-resistant vs. parental tumor cell pairs for the indicated proteins and phospho-proteins. **B.** Real time PCR analysis of ErbB family members. **C.** Western blot analysis of protein lysates from indicated tumor cell pairs for phosphorylated p38 (p-p38) and total p38. Bar graph represents the expression of p-p38 or p38 relative to GAPDH. [ER: erlotinib resistant].

To investigate the potential mechanism(s) involved in erlotinib resistance in our experimental model systems, expression of various kinases previously known to mediate tumor refractoriness to EGFR inhibition was evaluated. As shown in Figure 2A and 2B, EGFR protein and mRNA levels were slightly increased, decreased or remained unchanged in the erlotinib-resistant lines derived from HCC4006, H441, and A549 cells, respectively, compared to their corresponding parental cells. In addition to EGFR, the levels of mRNA encoding for the other members of the ErbB family, including ErbB2 and ErbB3, showed a decrease rather than an increase in all resistant tumor cell lines (Figure 2B). Amplification of the c-MET proto-oncogene has been previously linked to acquired resistance to EGFR kinase inhibitors [26]. In this study, c-MET expression levels were reduced (~50%) rather than increased in the H441 and A549 resistant vs. control cells (Figure 2A). No changes were detected in the expression or phosphorylation of AXL (Figure 2A), a kinase recently shown to be associated with erlotinib-resistance and acquisition of mesenchymal-like features in NSCLC [15].

**Figure 3:**
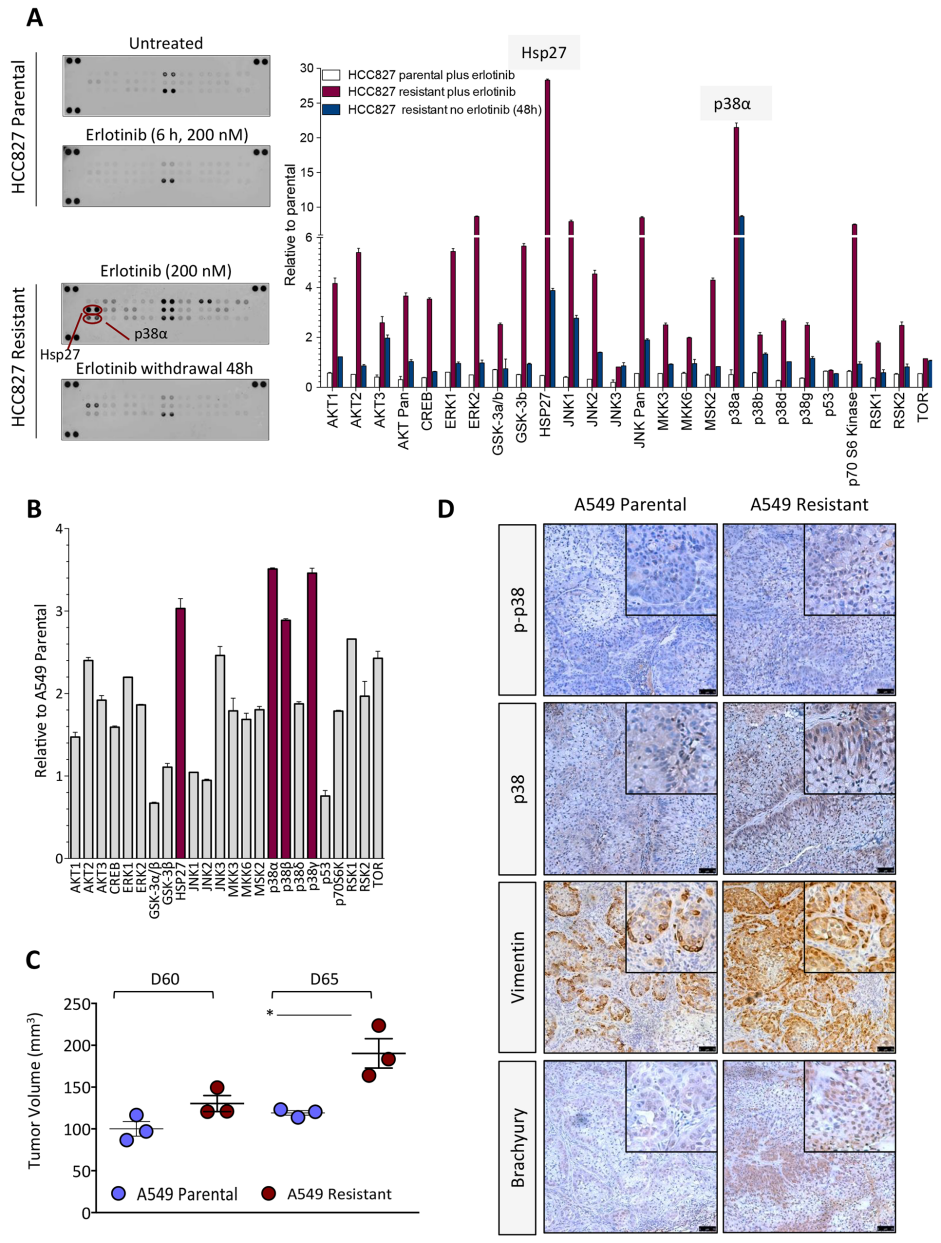
Kinase phosphorylation profiling in erlotinib-resistant cells **A.** Kinase phosphorylation profiling in HCC827 parental vs. erlotinib-resistant cells treated as indicated. Bar graph represents the expression of each phospho-kinase (relative to untreated parental cells) in indicated cells. **B.** Analysis of phospho-kinases and their normalized ratio in A549 erlotinib-resistant vs. parental cells. **C.** Growth of A549 cells (parental vs. resistant) as subcutaneous xenografts in nude mice. Shown is the tumor volume for individual mice at days 60 and 65 post-tumor implantation. **D.** Immunohistochemistry analysis of p-p38, p38, vimentin and brachyury expression in xenograft tumors of parental vs. erlotinib-resistant A549 cells.

As shown in Figure 2C, erlotinib-resistant cell lines demonstrated markedly increased levels of phosphorylated p38 (p-p38) mitogen-activated protein kinase (MAPK) compared to the parental cells. The expression of total p38 was also elevated in H441 and A549 erlotinib-resistant vs. parental lines. The substantial upregulation of p-p38 was subsequently studied with the HCC827 parental/resistant pair. Utilizing an antibody array for detection of 24 human phospho-MAPK kinases, enhanced phosphorylation of multiple MAPK was observed in the erlotinib-resistant HCC827 cells maintained in the presence of erlotinib (Figure 3A, red bars) vs. parental cells. The two kinases that exhibited the highest degree of upregulation in erlotinib-resistant vs. parental HCC827 cells were the heat shock protein 27 (Hsp27) and p38-alpha (Figure 3A). These kinases remained highly upregulated in HCC827 resistant cells following 48-hour erlotinib withdrawal. Hsp27, a protein that facilitates refolding of damaged proteins in response to stress conditions, has been shown to mediate resistance to apoptosis, in particular in response to cytotoxic drugs [27]. Interestingly, Hsp27 is a downstream target of the p38 kinase [28], a result that further supports our observations regarding enhanced p38 kinase activity in the erlotinib-resistant cells. In subsequent studies, a phospho-kinase array analysis was also performed with the A549 parental vs. erlotinib-resistant pair. As shown in Figure 3B, Hsp27 and the p38-alpha, -beta and -gamma MAPK were the most phosphorylated kinases in erlotinib-resistant vs. parental A549 cells. Studies conducted *in vivo* with tumor xenografts of A549 parental and erlotinib-resistant cells (Figure 3C) demonstrated the sustained overexpression of p-p38 and total p38 kinase, as well as overexpression of the mesenchymal marker vimentin and the EMT-associated transcription factor brachyury (Figure 3D). Thus, the results with the experimental models analyzed here indicate that elevated expression of p38 and its phosphorylated form is a central feature in the context of acquired erlotinib resistance.

### Acquired resistance to erlotinib is associated with activation of the IL-8/IL-8R axis

**Figure 4:**
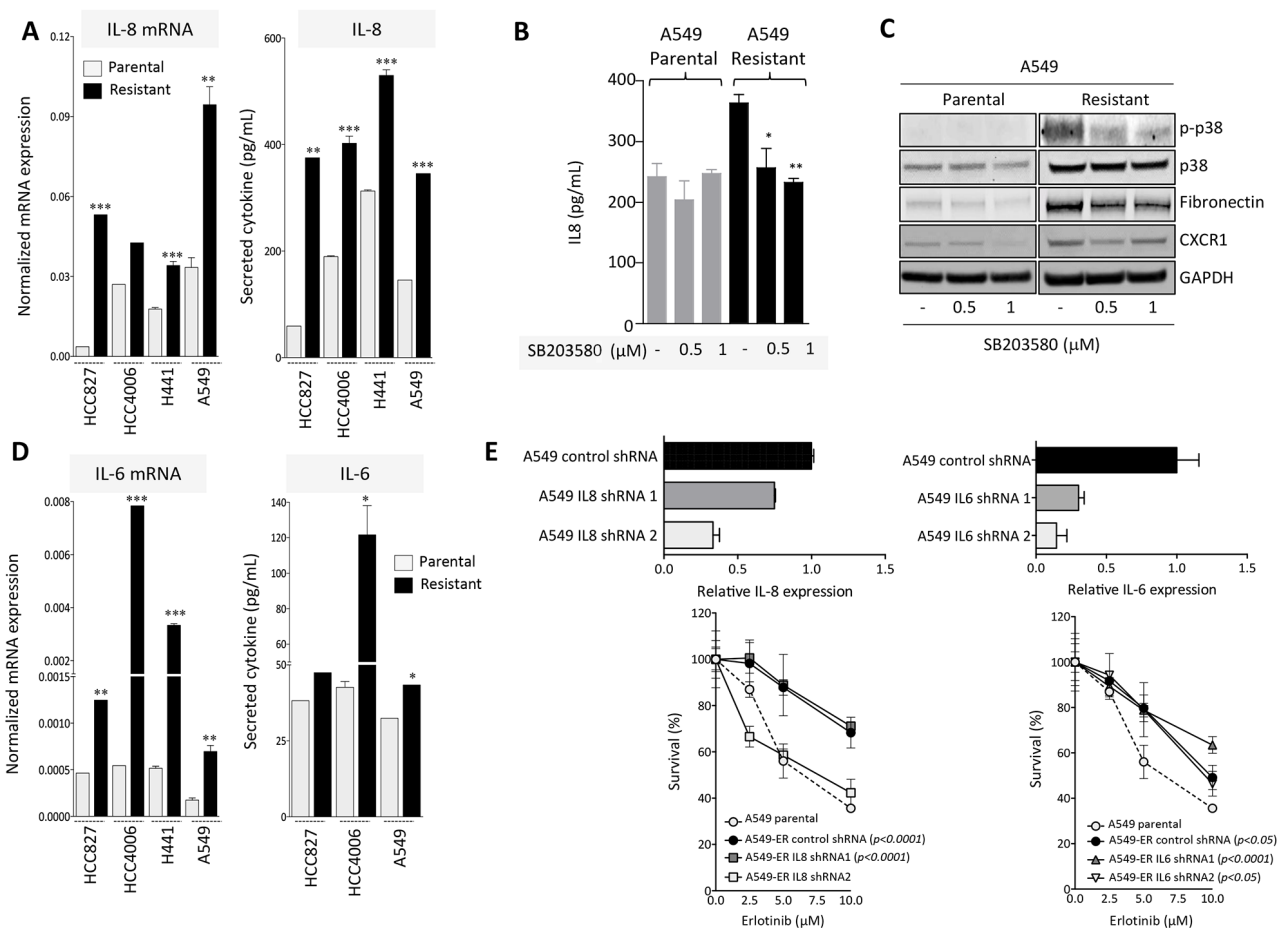
IL-8 signaling is upregulated in erlotinib-resistant cells **A.** IL-8 expression in erlotinib-resistant vs. parental cell lines measured at the mRNA (left) and secreted protein levels (right). **B.** IL-8 secretion in culture supernatants of A549 parental vs. erlotinib-resistant cells left untreated or treated *in vitro* with indicated doses of the p38 inhibitor SB203580. **C.** Western blot analysis of protein lysates from indicated tumor cells treated with the p38 inhibitor. **D.** IL-6 expression in erlotinib-resistant vs. parental cell lines measured at the mRNA (left) and secreted protein levels (right). **E.** *Upper panels*: expression of IL-8 or IL-6 in A549 cells transfected with IL-8 or IL-6 targeting shRNA constructs (1 and 2), relative to A549 cells transfected with a control non-targeting shRNA. *Bottom panels*: survival of indicated tumor cells in response to various doses of erlotinib. Indicated *p* values were calculated by two-way ANOVA relative to A549 parental cells.

In a previous study we have demonstrated a central role for the inflammatory cytokine IL-8 in the induction and maintenance of mesenchymal traits in epithelial cancer cells [23]. Recent clinical evidence suggests that the expression of IL-8 is an unfavorable prognostic factor in various types of carcinomas, including NSCLC [29]. In the present study it was further investigated whether the IL-8/IL-8R axis could also be implicated in the development of erlotinib resistance in lung carcinoma cells. As shown in Figure 4A, erlotinib-resistant HCC827, HCC4006, H441 and A549 cells displayed significantly higher levels of IL-8 mRNA and IL-8 secreted protein than their corresponding control cells. Additionally, H441 and A549 erlotinib-resistant cells demonstrated enhanced expression of the IL-8 receptor alpha (CXCR1) when compared to the parental cells (Supplementary Figure S1). These results indicated that mesenchymal-like cells generated in the context of erlotinib resistance have upregulated the IL-8/IL-8R signaling loop, which, in turn, could be responsible for the acquisition and/or maintenance of mesenchymal traits in those cells. The results are also in agreement with a recent report demonstrating the significant upregulation of IL-8 in gefitinib-resistant, EGFR mutated lung cancer cells [30].

Next, to investigate whether enhanced p38 signaling has any relevance on the upregulation of IL-8 in erlotinib-resistant cells, A549 parental vs. resistant cells were treated with the p38-specific small molecule inhibitor SB203580 prior to assessing IL-8 levels in culture supernatants. Inhibition of p38 kinase was able to substantially decrease the levels of secreted IL-8 to levels observed with parental A549 cells, validating the importance of p38 in this system (Figure 4B). In addition, expression of p-p38, CXCR1 and mesenchymal fibronectin were markedly reduced in A549 erlotinib-resistant tumor cells pre-treated with the p38 kinase inhibitor (Figure 4C), suggesting that blockade of p38 could alleviate mesenchymal features that, in turn, may contribute to tumor resistance.

Since various studies have now indicated an important role for the IL-6/STAT3 axis as a mediator of resistance to EGFR inhibition in lung adenocarcinomas [31, 32], we have also analyzed whether IL-6 was upregulated in the cell models utilized here. All resistant cell lines showed a significant upregulation of IL-6 compared to the parental counterparts, particularly at the mRNA level (Figure 4D). These results prompted us to compare the potential role of IL-6 vs. IL-8 in mediating resistance to erlotinib in the A549 model. Expression of IL-8 or IL-6 was silenced in A549 erlotinib-resistant cells, (Figure 4E, upper panels) and the ability of these cells to withstand treatment with various doses of erlotinib was subsequently assayed. As shown in Figure 4E, efficient silencing of IL-8 expression in A549-IL8 shRNA2 (compared to control shRNA cells) reconstituted erlotinib sensitivity to the levels observed with parental cells (Figure 4E, left bottom panel). This effect was not observed in cells transfected with IL8 shRNA1 that demonstrated only a small reduction on the levels of IL-8. In contrast, both IL6 shRNA constructs (1 and 2) markedly reduced IL-6 levels in transfected A549 erlotinib-resistant cells, but failed to reconstitute erlotinib sensitivity (Figure 4E, right bottom panel). These results indicate that in the model evaluated here, IL-8 and not IL-6 mediates acquired resistance to erlotinib.

### Blockade of IL-8 signaling alleviates resistance to erlotinib

**Figure 5:**
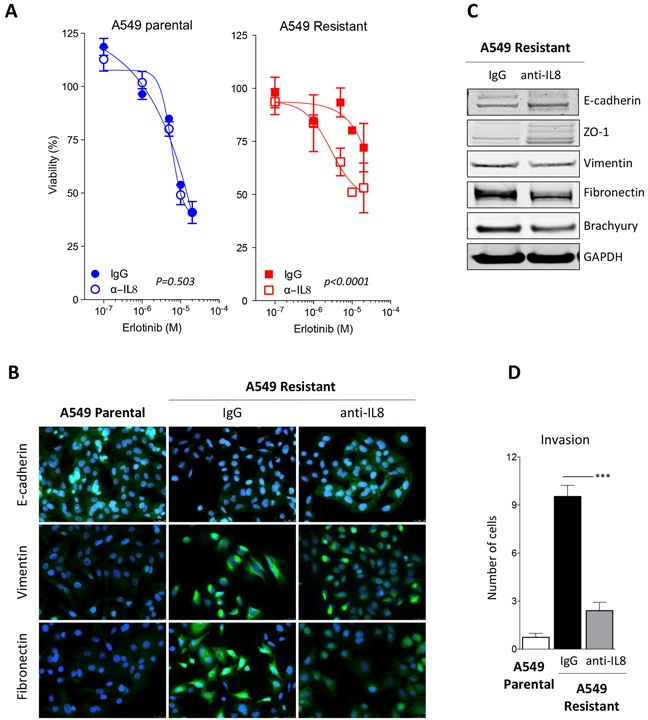
Blockade of IL-8 signaling reverts erlotinib resistance **A.** Assessment of erlotinib response in parental vs. erlotinib-resistant A549 cells previously treated with control IgG or neutralizing anti-IL-8 antibody [*p* values calculated by two-way ANOVA]. **B.** Immunofluorescent analysis (green signal) of epithelial E-cadherin and mesenchymal vimentin and fibronectin in A549 parental vs. erlotinib-resistant cells treated with a control IgG or a neutralizing anti-IL-8 antibody. Blue signal corresponds to DAPI stained nuclei. **C.** Western blot analysis of indicated markers in A549 erlotinib-resistant cells treated with control IgG or anti-IL-8 antibody. **D.** Invasion assay performed with indicated cells treated with control IgG or anti-IL-8 antibody prior to the assay.

To further evaluate the potential role of the IL-8/IL-8R axis on the acquired resistance to erlotinib and its associated mesenchymal-like phenotype, blocking experiments were conducted with a neutralizing antibody against IL-8. A549 erlotinib-resistant cells pretreated *in vitro* with anti-IL-8 regained erlotinib sensitivity to levels comparable to those of A549 parental cells (Figure 5A). Similar results were obtained with the H441 tumor cell pair (data not shown). In addition to improving response to erlotinib treatment, a significant reemergence of epithelial features, including a marked upregulation of epithelial E-cadherin and ZO-1 and the loss of mesenchymal vimentin and fibronectin expression was observed when A549 erlotinib-resistant cells were cultured in the presence of neutralizing anti-IL-8 (Figures 5B, 5C). As previously shown with breast cancer models [23], blockade of IL-8 signaling resulted in a substantial decrease of brachyury protein expression (Figure 5C) and reduced invasive properties (Figure 5D), adding more evidence of the mesenchymal-epithelial switch taking place in the absence of IL-8 signaling.

### Blockade of IL-8 signaling can sensitize erlotinib-resistant cells to chemotherapy

**Figure 6:**
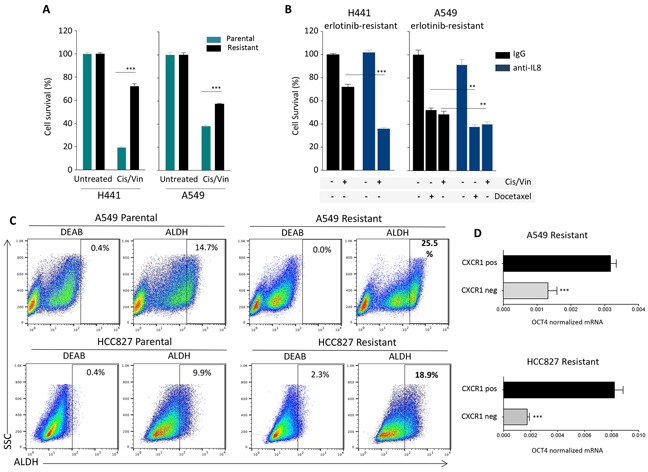
IL-8-dependent chemotherapy resistance and stemness **A.** Survival of H441 and A549 tumor cell pairs (parental vs. erlotinib-resistant) in response to treatment with cisplatin and vinorelbine combination in culture. **B.** Survival of erlotinib-resistant H441 and A549 cells in response to treatment with cisplatin and vinorelbine or docetaxel. Tumor cells were cultured in the presence of control IgG or neutralizing anti-IL-8 antibody for 72 hours prior to the chemotherapy treatment. **C.** Analysis of Aldehyde Dehydrogenase (ALDH) activity using the Aldefluor Kit (Stemcell) in indicated tumor cell pairs. The ALDH inhibitor Diethylaminobenzaldehyde (DEAB) was used to control for background fluorescence. **D.** Expression of mRNA encoding for the stem cell-associated marker OCT4 inCXCR1 positive vs. negative fractions isolated from A549 and HCC827 erlotinib-resistant cells, relative to GAPDH.

We next investigated whether the acquisition of resistance to erlotinib in the NSCLC cells could concomitantly drive resistance to chemotherapy. As shown in Figure 6A, erlotinib-resistant, mesenchymal H441 and A549 cells showed a significant survival advantage when challenged with a combination of the chemotherapy agents cisplatin and vinorelbine, compared to their parental cell counterparts. To investigate whether resistance to chemotherapy induced by acquisition of erlotinib resistance could be alleviated by blockade of the IL-8 signaling axis, H441 and A549 erlotinib-resistant cells were pre-treated in culture with a neutralizing anti-IL-8 antibody and subsequently exposed to the combination cisplatin/vinorelbine or docetaxel. Blockade of IL-8 signaling significantly improved the cytotoxic effect of the chemotherapeutic drugs (Figure 6B), reinforcing the idea that activation of the IL-8 axis in erlotinib-resistant cells imparts multidrug resistance traits, potentially due to their acquisition of a more mesenchymal-like tumor phenotype.

As the ability to withstand cytotoxic treatments is a known feature of stem cells, and in light of recent reports associating the occurrence of EMT with tumor stemness, we analyzed the A549 and HCC827 cell pairs for the presence of cells with high levels of aldehyde dehydrogenase (ALDH) activity, which has been previously reported to be increased in cells with precursor features (i.e., stem cells and stem-like cells). As predicted, both A549 and HCC827 erlotinib-resistant cell lines exhibited greater percentage of ALDH bright cells (25.5 and 18.9%, respectively, Figure 6C), compared to their corresponding parental lines (14.7 and 9.9% for A549 and HCC827, respectively). This data was in agreement with previous reports demonstrating the enrichment of stem-like populations in tumor cells selected for resistance to EGFR inhibition in long-term culture [30]. To further investigate whether upregulation of the IL-8 axis could be related to the presence of higher percentage of cells bearing a stem-like phenotype, erlotinib-resistant A549 and HCC827 cells were separated into CXCR1 positive and negative fractions by using magnetic beads, and the levels of the stem cell marker *OCT4* were evaluated by real time PCR analysis in each cell fraction. As shown in Figure 6D, there was a significant increase of *OCT4* mRNA levels in the CXCR1 positive vs. negative fraction, thus reinforcing the observations that IL-8 signaling is enhanced in stem-like cell populations enriched in the EGFR-resistant cell lines [30].

## DISCUSSION

We have identified the activation of the IL-8/IL-8R axis in NSCLC cells as a novel mechanism involved in the acquisition of resistance to targeted inhibition of the EGFR kinase. Acquisition of resistance to erlotinib was shown to be concurrent with the modulation of tumor phenotype towards a mesenchymal-like state (i.e., EMT), as well as resistance to conventional chemotherapies. Blockade of the IL-8 signaling axis via the use of a neutralizing antibody directed against IL-8 resulted in the re-acquisition of epithelial features, sensitization to EGFR inhibition and re-establishment of cytotoxic responses to chemotherapy treatment, thus providing evidence for a potential approach to enhance clinical responses to EGFR TKIs in the clinic.

Inhibition of the EGFR kinase via the use of small molecule inhibitors, including erlotinib and gefitinib, constitutes the first line of therapy for patients whose tumors harbor sensitizing mutations in the kinase domain of the EGFR, or the second or third line of treatment for patients with wild type EGFR. Here we have investigated possible mechanisms of tumor resistance to EGFR inhibition in both EGFR mutant and wild type NSCLC cells in order to optimize the use of small molecule therapies directed against the EGFR in a broad range of patients. The tumor cell lines with wild type EGFR evaluated here, H441 and A549, harbor a mutated version of the KRAS proto-oncogene, a genetic feature with 30% prevalence among lung adenocarcinomas. Unlike colon carcinoma, where tumors harboring mutant KRAS are known to be resistant to EGFR inhibition [33], the significance of KRAS mutations upon the benefit of anti-EGFR therapies in patients with lung cancer remains unclear [34]. Our results demonstrated that long-term exposure of NSCLC cells to erlotinib results in the acquisition of tumor phenotypic features associated with EMT, a phenomenon that was more prominent with KRAS-mutated cell lines. This was demonstrated by the substantial upregulation of markers typically associated with the mesenchymal phenotype, including fibronectin, vimentin, and N-cadherin, coupled with the loss of epithelial cell contacts mediated by the junction protein E-cadherin in tumor cells resistant to erlotinib.

The acquisition of EMT features by tumors resistant to EGFR inhibition has been previously described both in preclinical studies and with clinical samples [35]. For example, the analysis of expression of epithelial and mesenchymal markers in multiple NSCLC lines has revealed a negative correlation between the occurrence of tumor EMT and the cytotoxic response to EGFR inhibition *in vitro* and in xenografts [16]. The relevance of EMT in resistance to EGFR inhibition in the clinical setting was validated by the demonstration that a 76-gene EMT signature developed *in vitro* with NSCLC lines could then predict response to erlotinib in patients with EGFR/KRAS wild type tumors [36]. Moreover, Sequist et al. [37] demonstrated the occurrence of EMT in biopsies from some patients with drug-resistant, EGFR mutant tumors. We have previously shown that human epithelial breast cancer cells undergoing an EMT via overexpression of brachyury markedly upregulate the expression of IL-8 and its receptors, CXCR1 and CXCR2 [23]. In addition, we have shown that antibody blockade of IL-8 receptors in mesenchymal-like tumor cells can efficiently revert their phenotype towards an epithelial one, therefore providing evidence that the IL-8/IL-8R axis is essential for the maintenance of the phenotype of mesenchymal-like cancer cells [23]. Others have also shown the existence of a link between IL-8 and tumor EMT; in colorectal cancer cell lines, for example, the induction of EMT via incubation with TGF-β [38] or via SNAIL overexpression [39] has been shown to induce the secretion of IL-8. In agreement with those previous reports, we have demonstrated here a robust increase of IL-8 secretion and upregulation of CXCR1 in erlotinib-resistant lung cancer cells, compared to their parental counterparts, regardless of the mutational status of EGFR or KRAS. The chemokine IL-8 plays multiple roles as an inflammatory cytokine by mediating the activation and chemotaxis of various immune cells [40]. In addition, IL-8 can be secreted by cancer cells, particularly under stress conditions such as hypoxia or exposure to chemotherapy agents [41, 42], and potentially facilitate various aspects of tumor growth and progression, including angiogenesis, proliferation, survival, migration, and EMT [43, 44]. In the context of erlotinib resistance, a robust upregulation of the IL-8 axis is expected to favor tumor migration and dissemination, as well as to facilitate therapeutic unresponsiveness via the establishment and maintenance of an EMT.

The control of IL-8 gene expression is achieved both at the transcriptional and post-transcriptional levels in response to external stimuli [45]. We have shown that erlotinib-resistant cells have a marked upregulation of the cytokine/stress-inducible p38 MAPK and its phosphorylated form, which is known to regulate IL-8 secretion by mediating the stabilization of IL-8 mRNA [45, 46], and that blockade of p38 via a small molecule inhibitor is able to reduce the secretion of IL-8 in erlotinib-resistant cells while reversing features of EMT. In order to understand the potential mechanism of activation of p38, we have simultaneously evaluated the expression of multiple phospho-MAPKs in the HCC827 and A549 tumor cell pairs. In HCC827 erlotinib-resistant cells, phosphorylation of p38 alpha was predominant over that of the variants p38 beta, delta and gamma. In addition, Hsp27, a target of p38 MAPK and a mediator of resistance to cell death was remarkably upregulated in HCC827 erlotinib-resistant cells, thus indicating the increased activity and functionality of p38 in these cells. Activation (≥ 2-fold) of MKK3 and MKK6, both dual kinases known to activate the various p38 isoforms via phosphorylation, was also observed in these cells (Figure 3A). In A549 erlotinib-resistant cells, activation of the p38 alpha, beta and gamma isoforms (> 2-fold) was observed, though phosphorylation of MKK3 and MKK6 was only modest (~1.7-fold) in erlotinib-resistant vs. parental A549 cells (Figure 3B). These findings indicate that MKK3/6 might be responsible for the activation of p38 MAPK in the erlotinib-resistant models analyzed here.

In the context of erlotinib resistance, we demonstrated that blockade of IL-8 signaling via the use of a neutralizing antibody can revert mesenchymal tumor features and re-sensitize tumor cells to the cytotoxic effect of erlotinib. This observation was further expanded to the ability of IL-8 blockade to revert chemotherapy resistance that is also prominent in erlotinib-resistant cells. Our results also showed that IL-6 is markedly upregulated in erlotinib-resistant cells. However, loss of function experiments conducted with shRNAs specific for IL-8 vs. IL-6 demonstrated that IL-8 is involved in the resistance of the tumor cells to erlotinib, as silencing of IL-8 (but not IL-6) was able to reconstitute sensitivity to erlotinib to the level observed with parental cancer cells. Several previous reports have indicated a role for IL-8 signaling in tumor resistance to cytotoxic insults. For example, the silencing of the CXCR2 receptor in murine mammary tumor cells was shown to enhance antitumor activity of paclitaxel *in vivo* [47], and inhibition of CXCR2-mediated signaling in human colon cancer lines was shown to reduce cell migration and invasion while enhancing the tumor response to oxaliplatin [48]. The upregulation of IL-8 in response to EGFR inhibition has been previously observed in other systems; for example, head and neck cancer cells resistant to erlotinib showed enhanced IL-8 secretion in the context of EMT [49]. Our results confirm these previous observations on the role of IL-8 in resistance to EGFR inhibition and further expand them by demonstrating that upregulation of p38 MAPK signaling is responsible for enhanced IL-8 secretion in resistant tumor cells. Moreover, blockade of IL-8 could render erlotinib-resistant lung cancer cells more amenable to EGFR inhibition or chemotherapy. Resistance to chemotherapy is one of the features typically associated with tumor stemness. In agreement, here we demonstrate that erlotinib resistance associates with an increased percentage of ALDH bright cells. Moreover, in support of a potential link between stemness and activation of the IL-8 signaling axis, we also show that CXCR1 positive tumor cells isolated from erlotinib-resistant lines have significantly higher levels of the stem cell-related gene, *OCT4*. Although we have not performed experiments to demonstrate whether IL-8 directly plays a role as a driver of tumor stemness, a previous publication [30] has shown that silencing of IL-8 in gefitinib-resistant, EGFR-mutant lung cancer cells is able to significantly decrease the percentage of ALDH positive cells and to reduce the expression of various stemness related genes, including *NANOG, OCT4, SOX2,* and others.

Thus, inhibition of IL-8 signaling appears as an attractive potential intervention to augment the efficacy of EGFR inhibition approaches in lung cancer by preventing the establishment of EMT, and/or by reverting the phenotype of tumor cells that have undergone the EMT and became resistant to the effects of EGFR inhibitors. In addition, our results show that blockade of IL-8 signaling could circumvent resistance to chemotherapy that takes place concurrently with the acquisition of erlotinib resistance in lung cancer cells.

## MATERIALS AND METHODS

### Cell culture and generation of erlotinib-resistant cells

The following human lung carcinoma cell lines were obtained from the American Type Culture Collection (ATCC) and propagated as recommended: H441, A549 and HCC4006. HCC827 cells were kindly provided by Dr. U. Guha, NCI, NIH. The HCC4006 and HCC827 erlotinib-resistant cells were generated by exposing parental cells to increasingly higher doses of erlotinib (0.1 to 0.2μM for HCC827 and 0.05–0.4μM for HCC4006 cells), and subsequently expanded in media containing the highest applied dose of erlotinib. For establishment of H441 and A549 erlotinib-resistant cells, parental cells were exposed to a high dose of erlotinib (10-20μM) for 2 weeks, and subsequently expanded in media containing erlotinib 10μM. H441 and A549 cells (parental and derivatives) and parental HCC4006 and HCC827 cells were analyzed by short tandem repeat (STR) profile analysis for confirmation of their identity.

### Erlotinib and chemotherapy response

Cell growth and survival of tumor cells were evaluated by using 3-4,5-dimethylthiozol-2, 5-diphenyl-tetrazolium bromide (MTT, Sigma) or the CellTiter-Glo cell viability assay (Promega). Three-to-six replicates were used per condition, and all experiments were repeated at least two times; results are expressed as percentage viability relative to that of untreated (or DMSO treated) control cells. For evaluation of responses to chemotherapy, chemotherapy agents were added to adherent cell cultures for six hours at the following concentrations: cisplatin, 1000ng/ml; vinorelbine and docetaxel, 40ng/ml, followed by wash and addition of fresh media for indicated periods prior to evaluation of cell survival by the MTT assay.

### Real-time PCR

Total RNA was prepared using the RNeasy extraction kit (Qiagen) and reverse transcribed with the Advantage RT-for-PCR kit (Clontech). cDNA (10-50 ng) was amplified in triplicate using the Gene Expression Master Mix and the following TaqMan human gene expression assays (Applied Biosystems): *EGFR* (Hs01076077), *ErbB2* (Hs01001580), *ErbB3* (Hs00176538), *ErbB4* (Hs00955525), *IL-8* (Hs00174103), *IL-6* (Hs00985638) and *OCT4* (Hs00999632). Mean Ct values for target genes were normalized to mean Ct values for the endogenous control glyceraldehyde-3-phosphate dehydrogenase (GAPDH) [−ΔCt=Ct (GAPDH)-Ct (target gene)]. The ratio of mRNA expression of target gene vs. GAPDH was defined as 2^−ΔCt^. A Human EMT RT^2^ Profiler PCR Array (Qiagen) was used to evaluate expression of 84 genes involved in EMT on cDNAs prepared from HCC827 cells (parental vs. resistant), following the manufacturer's recommendations.

### Western blot

Cells were lysed in RIPA Lysis buffer (Santa Cruz Biotech) or 1X Blue loading buffer (Cell Signaling). Protein lysates (25-100μg) were resolved on SDS-PAGE and transferred onto nitrocellulose membranes using a standard western blot protocol. Membranes were probed with primary antibodies against E-cadherin, N-cadherin, fibronectin, vimentin, ZO-1, EGFR, p-EGFR, (BD Biosciences), CXCR1 (Thermo Scientific), GAPDH (Santa Cruz), AXL, p-AXL (R&D Systems), p38, p-p38 (Cell Signaling), c-MET (Invitrogen) and brachyury (MAb 54-1) [50], at 4°C overnight. Membranes were incubated with an appropriate secondary antibody conjugated with IRDye and detected by the Odyssey infrared detection method (Li-COR Biotechnology). For detection of phospho-MAPK in tumor lysates of HCC827 and A549 tumor cell pairs, a Proteome Profiler Human Phospho-MAPK Array Kit (R&D Systems) was used, following the manufacturer's recommendations. Signal was detected and quantified by the Odyssey infrared detection method (Li-COR Biotechnology).

### Immunofluorescence and immunohistochemistry

Cells cultured on glass cover slips were fixed with 4% formaldehyde, permeabilized with 0.2% Triton-X and blocked with PBS containing 10% goat serum and 1% FBS. Cover slips were incubated overnight with primary antibody against E-cadherin, fibronectin, vimentin (BD Biosciences, 1:50 dilution) prepared in 1% FBS in PBS 1X, and subsequently washed and incubated with an Alexa Fluor-488 goat anti-mouse antibody (Invitrogen) for 1hr at room temperature. Cover slips were mounted using VECTASHIELD with DAPI mounting medium (Vector Laboratories). Images were captured utilizing a Leica Fluorescent microscope. Immunohistochemistry analysis was carried out as previously described [50]; the antibodies used were anti-p38 and p-p38 (Cell Signaling), anti-vimentin (GeneTex) and anti-brachyury (MAb 54-1).

### IL-8 or p38 signaling blockade

For IL-8 blockade, cells were exposed to 10μg/mL of a commercially available neutralizing anti-IL-8 antibody [MAB208] or control IgG (R&D Systems) for 72 hours prior to indicated experiments. For p38 signaling blockade, cells were exposed to 0.5-1μM SB203580 (Selleckchem) for 72 hours prior to indicated experiments.

### Invasion assays

Invasion assays were performed in Blind Well Chambers (Neuroprobe). Briefly, RPMI-1640 medium supplemented with 20% FBS was added to the lower chambers and 1-3 × 10^5^ cells in serum-free medium were added onto the upper chambers. After 24 to 48 hours incubation at 37°C, filters were fixed and stained with Diff-Quik stain (Siemens) and the number of cells attached to the bottom side of the filters was counted and averaged from 5 random 100X microscope objective fields. Experiments were conducted in triplicate samples for each cell line and repeated at least two times.

### Tumor xenograft studies

To establish subcutaneous tumors with the A549 pair, 5-6 week old female athymic nude (*nu/nu*) mice (The Jackson Laboratory) were inoculated with 4×10^6^ cells in 100μL of HBSS admixed with Matrigel 50% (v/v). All mice were housed and maintained in micro isolator cages under specific pathogen-free conditions and in accordance with the Association for Assessment and Accreditation of Laboratory Animal Care (AAALAC) guidelines. All experimental studies were carried out under approval of the NIH Intramural Animal Care and Use Committee. Tumor volume was recorded; at the completion of the experiment, tumors were harvested and processed for immunohistochemical evaluation.

### Statistical methods

Data were analyzed using GraphPad Prism (GraphPad Software) using a two-sample Student's *t*-test, unless indicated. Data points in graphs represent the mean ± SEM; [*p<0.05; ** *p*<0.01; *** *p*<0.001].

## SUPPLEMENTARY FIGURE


