# Supplementary material for: IL-8 signaling is involved in resistance of lung carcinoma cells to erlotinib
Source: Oncotarget. 2016 May 27;7(27):42031–44. doi: 10.18632/oncotarget.9662 (PMC5173114; doi:10.18632/oncotarget.9662)
Supplement: Supplementary file 1 [file oncotarget-07-42031-s001.pdf]

IL-8 signaling is involved in resistance of lung carcinoma cells to erlotinib

SUPPLEMENTARY FIGURE

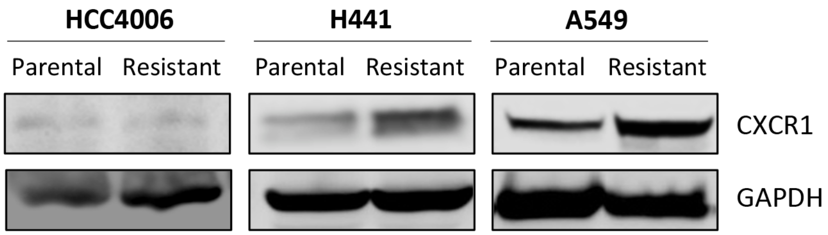

Supplementary Figure S1: IL-8 signaling is upregulated in erlotinib-resistant cells. Western blot analysis of protein lysates from indicated tumor cell pairs for expression of the IL-8 receptor alpha (CXCR1).
